# Johnny Depp, Reconsidered: How Category-Relative Processing Fluency Determines the Appeal of Gender Ambiguity

**DOI:** 10.1371/journal.pone.0146328

**Published:** 2016-02-04

**Authors:** Helen E. Owen, Jamin Halberstadt, Evan W. Carr, Piotr Winkielman

**Affiliations:** 1 Department of Psychology, University of Otago, Dunedin, New Zealand; 2 Department of Psychology, University of California San Diego, La Jolla, California, United States of America; 3 Behavioural Science Group, Warwick Business School, University of Warwick, Coventry, United Kingdom; 4 Department of Psychology, University of Social Sciences and Humanities, Warsaw, Poland; Brock University, CANADA

## Abstract

Individuals that combine features of both genders–gender blends–are sometimes appealing and sometimes not. Heretofore, this difference was explained entirely in terms of sexual selection. In contrast, we propose that part of individuals’ preference for gender blends is due to the cognitive effort required to classify them, and that such effort depends on the context in which a blend is judged. In two studies, participants judged the attractiveness of male-female morphs. Participants did so after classifying each face in terms of its gender, which was selectively more effortful for gender blends, or classifying faces on a gender-irrelevant dimension, which was equally effortful for gender blends. In both studies, gender blends were disliked when, and only when, the faces were first classified by gender, despite an overall preference for feminine features in all conditions. Critically, the preferences were mediated by the effort of stimulus classification. The results suggest that the variation in attractiveness of gender-ambiguous faces may derive from context-dependent requirements to determine gender membership. More generally, the results show that the difficulty of resolving social category membership–not just attitudes toward a social category–feed into perceivers’ overall evaluations toward category members.

## Introduction

Gender is fundamental dimension on which people categorize themselves and others [1]. For most of recorded history, reporting one’s “gender” involved a dichotomous categorization of “male” or “female”, but recently the concept has become more diffuse, referring as much to a person’s self-concept and self-presentation as to their biology or sexual orientation [2]. For instance, Facebook allows users to choose among 56 different gender designation (e.g., pangender, bigender, transgender, etc.) and 10 possible gender combinations [3].

Social norms, however, have advanced faster than psychological ones. Perceivers, not to mention legal and social institutions, continue to judge gender dichotomously [1–2,4], and violations of traditional gender norms are punishable by prejudice, ostracism, and even violence [5]. In particular, perceivers attend early to gendered facial features and compare them against male and female prototypes, with implications for categorization and judgment [6–7]. Thus, regardless of perceivers’ explicit acceptance of the graded nature of gender categories, perceivers still assess how well a face matches physical male/female prototypes.

Moreover, the implications of counter-normative appearance may vary with nature of the norm being violated. There is limited research on “masculine looking” women, but extant findings indicate that such individuals are disadvantaged compared to women with more “feminine” features. Male and female perceivers show a consistent preference for feminine-looking women [8–10], with masculine features associated with dominance and perceived as threatening or competitive, particularly by women low in dominance themselves [11–13].

On the other hand, *men* who appear with *feminine* features appear to elicit more variable reactions, which have also received more research and attention in pop-culture. On the one hand, “feminine” men, contrary to masculine women, are often noted for their beauty. Researchers have even coined a term for the attractiveness of men with feminine features–the “Johnny Depp effect” [14]–with reference to the movie star’s large eyes, small jaw, and high-arched eyebrows (features he shares with other male celebrities such as singers and actors like Justin Bieber, Harry Styles, Orlando Bloom, and Leonardo DiCaprio). Laboratory studies that digitally enhance gendered features confirm that feminized faces are generally more attractive than masculinized ones [9,15], and meta-analyses have revealed cross-cultural preferences for these feminized faces over sex-typical faces, independent of the faces’ race [9,15–17].

In other situations, however, feminine men, like masculine women, are scorned. Feminine-looking men are in some cases perceived as less competent, less suitable for “masculine” roles (e.g., “team leader”) [18–19]. They are also seen as more likely to be homosexual and to suffer prejudices that accompany that attribution [20]. Li-Vollmer and LaPointe (2003) argue that relatively feminine features and behaviors are used to signal deviance in Disney villains [21]. Feminized men even perceive *themselves* as less dominant [22–23], and as a result, overestimate the dominance of their male rivals to reduce threat, injury, and loss of resources [13].

Thus, gender ambiguous *men*, at least, are sometimes seen as attractive (or favorable on other dimensions), and sometimes seen as unattractive or judged negatively compared to men exhibiting extreme sex-typical traits. Previously, these discrepant findings have been interpreted in exclusively evolutionary psychological terms [24–26]. In these accounts, both masculine and feminine features in men signal reproductively valuable traits, but on different dimensions. Masculine facial features in men signal valuable physical traits such as strength, health, and reproductive potential [27], whereas feminine facial features in men signal valuable *social* traits such as empathy, warmth, and cooperation [9], which are associated with a man’s long-term commitment to his offspring.

Put bluntly, feminine-looking men may father genetically inferior offspring, but may be more committed to raising them, such that both types of men are appealing to mates in different individual or societal conditions. Consistent with this analysis, female perceivers’ preference for feminine men has been shown to depend on their level of commitment to their partner [8] and the phase of their menstrual cycle [28–29]. Women favoring long-term versus short-term relationships show a preference for feminine and masculine traits, respectively, and have better memory for encounters with men displaying such traits [8,30]. Interestingly, DeBruine et al. (2010) has found that the preference for feminized men increases with the general physical health of a society, presumably reflecting an evolutionary tradeoff in mate choice [31].

Although such data hint at the possible ultimate functions of our reactions to feminized male faces, they provide limited insight into the proximate mechanism(s) responsible for these reactions. Though the exact proximate mechanism is rarely discussed in evolutionary explanations, it presumably involves differential sensitivity to reproductively valuable traits as a function of mating and subsequent parenting needs. In contrast, we propose that part of individuals’ evaluative reactions to such faces, and perhaps to gender-ambiguous faces in general, can be accounted for by the cognitive effort required to classify them, and that such effort will depend on the context in which a blend is judged. This is because such faces are *atypical*, in the technical sense of being dissimilar to male and female prototypes. Posner and Keele (1968) first documented the relationship between stimulus typicality and processing difficulty, showing that dot-pattern distortions were increasingly difficult to classify as they deviated from the prototype from which they were generated [32]. This relationship has since been generalized to many other types of categories, including ad hoc [33] and even formal categories (e.g., odd numbers) [34]. More importantly, subsequent research has shown that processing difficulty–termed *disfluency–*is associated with negative affect. Winkielman, Halberstadt, Fazendeiro, and Catty (2006) found, in a replication of Posner and Keele (1968), that as dot patterns increasingly deviated from their group prototype, they were not only harder to classify, but were also liked less, and that the first effect (disfluency of deviations) partially explained the second (dislike of deviations) [32,35]. Indeed, any factor that impedes the perception, classification, or processing of a stimulus tends to produce more negative evaluations of that stimulus on a variety of social dimensions and measures, including intelligence [36], attractiveness and trustworthiness [37], liking [38], physiological smiling responses [39], and even on some economic indicators such as brand evaluations [40] or stock purchases [41].

Applied to gender ambiguity, this disfluency account predicts that faces that blend gender features will be judged negatively. However, this should only happen when such blends first need to be classified in terms of their gender. This is because human faces (like all stimuli) can be categorized along multiple dimensions, so while gender blends are unusual “men” and “women”, they can be perfectly usual on other stimulus categories (e.g., when judged as faces in general, on race, age, emotion, etc.). Therefore, we predict that gender-ambiguous individuals should only be disfluent, and therefore relatively unattractive, when they are classified *as* men and women, and not necessarily when the perceiver focuses on other dimensions where gender blends do not cause any classification disfluency.

Recent evidence suggests that blends of social categories vary in their appeal, depending on the effort needed to determine category memberships [37,42–44]. In one of the initial studies, Halberstadt and Winkielman (2014) first gave participants an opportunity to learn about faces from two families (“Acks” and “Blubs”) [44]. Later, participants took longer to classify morphs that were blends across the families and found them less attractive than prototypical members of each family. Critically, this devaluation of blends occurred only when the attractiveness rating required faces to be classified by family, but not when participants simply rated their attractiveness. Though not tested directly, the presumption was that the classification effort produced negative affect that generalized to the appeal of the blended face itself.

Applying this logic to the question of gender-ambiguous faces, we propose that the appeal of male-female blends depends on the effort involved in assigning them to social categories. Feminized male faces, by definition, represent poor examples of the “male” and “female” categories compared to pure (unblended) male and female faces. Therefore, when they are judged relative to gender specific categories, these blends should be relatively difficult to process, and in turn less appealing, with the second effect statistically explained by the first.

The present studies test these hypotheses by manipulating both gender ambiguity and, independently, the difficulty of categorization. In Study 1, participants were presented with sets of male-female morphs and classified each face in terms of its gender (or performed a control task), prior to rating the face’s attractiveness. We expected that, in the gender classification condition only, ambiguous faces would be rated as less attractive than unambiguous faces, producing a “U-shaped” morph-attractiveness relationship. Furthermore, we predicted that gender classification time–a measure of fluency–would vary with gender ambiguity, and would mediate the attractiveness effects. Study 2 was a replication of Study 1, but using a more complex stimulus set to test whether relative dislike for gender-ambiguous faces occurs only when participants experience processing difficulty on the gender dimension, or whenever they pay more attention to the face.

## Study 1

### Method

#### Participants

Eighty-one first- and second-year psychology students at the University of Otago (33 male, 48 female) took part in the experiment in exchange for course credit. In both studies, sample size was set, based on previous work, at approximately forty participants per between-subjects condition.

#### Ethics statement

This project was approved by the ethics committee at the University of Otago. Participants provided written informed consent to participate in the studies

#### Stimuli

**Fig 1:**
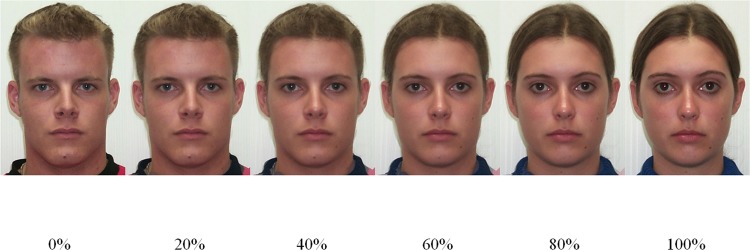
Examples of stimuli used in Study 1 as a percentage of the female parent.

Stimuli consisted of 110 gender-ambiguous faces, which were created by blending ten pairs of male and female faces from the University of Western Australia’s *Facelab* database. The original headshots were photographed against a white background in a uniformly lit room. Using Morpheus Photo Animation Suite software [45], we created a set of eleven faces from each pair: the two original “parents” and nine equally spaced blends, each representing a unique blend of male and female features (100% male, 90% male / 10% female, etc.), appearing 170 x 120mm on the screen. Examples of these morphs and their male and female parent faces appear in Fig 1.

#### Procedure

After giving informed consent, participants were randomly assigned to individual light- and sound-attenuated experimental cubicles. All stimuli were presented, and data collected, on iMac computers running Superlab software on 17-inch monitors. All participants rated all faces in a different random order on a 9-point Likert scale anchored at “not attractive” and “very attractive,” using their keyboard. However, prior to rating each face, half of the participants were instructed to categorize it as male or female, using corresponding keyboard keys, while control participants were instructed to press the spacebar as soon as they saw a face. In both conditions, a face was preceded by a 1000 ms fixation cross, and replaced by the attractiveness scale as soon as the participant responded (if a participant failed to respond within 2000 ms, a question mark appeared in the center of the screen to prompt them and remained on screen until they responded). The rating scale was removed as soon as a participant responded, and the next trial began after a 3000 ms inter-trial interval. The experiment took approximately 20 minutes and was run in conjunction with several other, unrelated studies, after which all participants completed a short demographics form and were debriefed.

### Results and Discussion

#### Analysis strategy

Data were analyzed at the level of the stimulus, aggregating across participants in each condition. Although intuitive, such analyses are potentially vulnerable to “noise” from random effects associated with participants, and to dependencies among particular stimuli with the same morph continuum. To verify that the results reported below are robust, we also analyzed the critical response time and attractiveness data using multilevel modeling (MLM) including random intercepts for participants and stimuli. The results of the MLM analyses, which replicate the results reported below, appear in the Supporting Information (S1 File).

Trials in which response times were shorter than 200 ms and greater than 2000 ms were excluded from analysis, and we excluded entirely data from eight participants who failed to classify at least 2/3 of the stimuli within these time constraints.

#### Manipulation check

To verify that the morphing procedure had the intended effect on the perception of the faces’ gender, the proportion of experimental participants classifying each image as female was regressed on the linear, quadratic, and cubic components of “femaleness” (the proportion of the female parent present in a morphed image, the squared proportion, and the cubed proportion). The results revealed significant linear and cubic effects (a classic pattern of categorical perception, with the greatest change in the middle third of the morph continuum) [46], β = 1.60 and -.73, *t*s = 26.63 and -12.16, *p*s < .001. The model explained 94% of the variance.

#### Fluency

**Fig 2:**
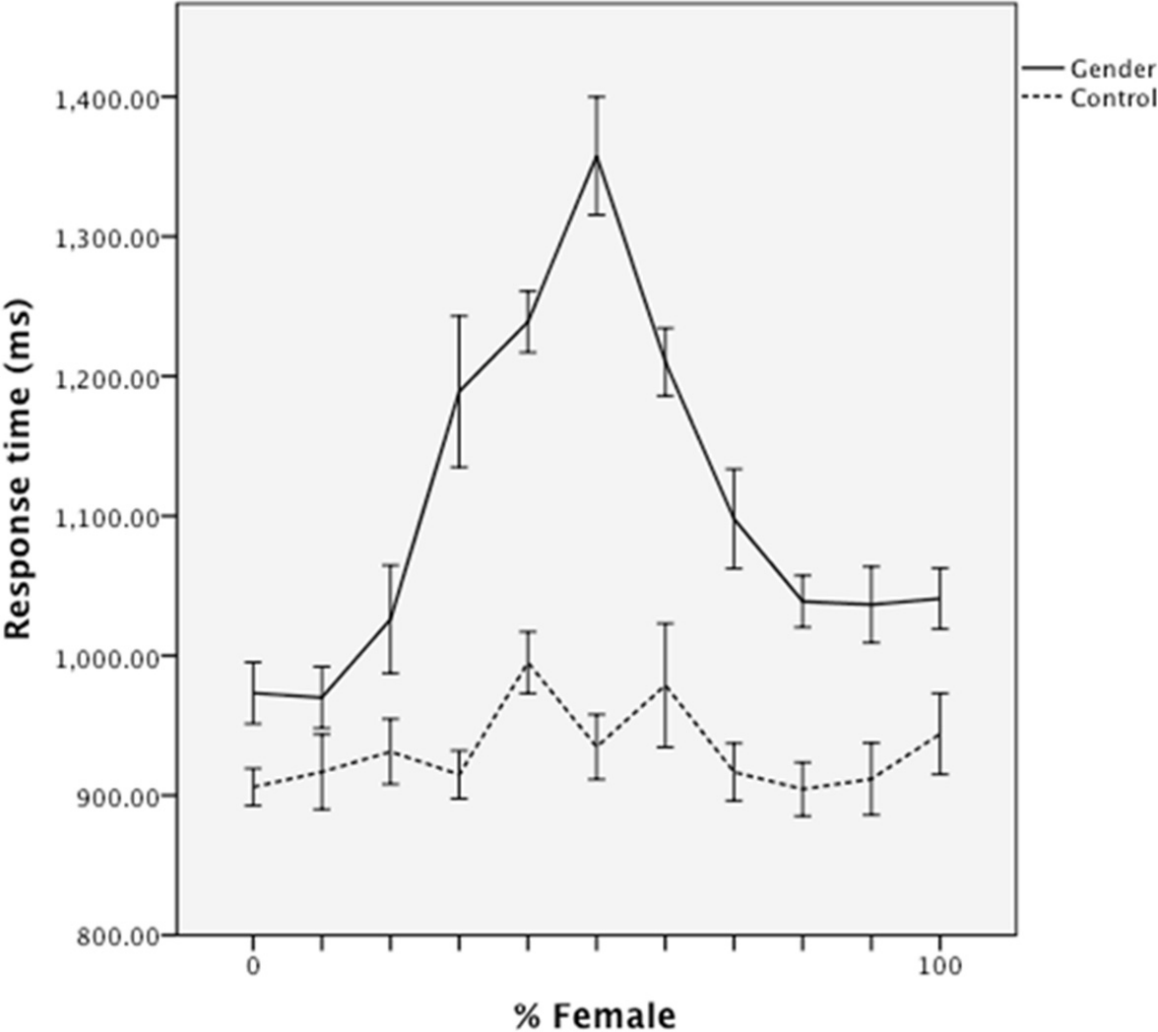
Mean classification response time as a function of morph level and classification group in Study 1. Error bars represent standard errors.

Processing fluency was operationalized, as in previous research, as classification time (i.e., in the control condition, classification as a “face”, in the gender condition, time to decide “male” or “female” prior to an attractiveness rating). These data were regressed on experimental condition (gender classification versus control); morph level and squared morph level (to model linear and quadratic effects, respectively); and both morph level by condition interactions, mean centered and entered simultaneously. The analysis revealed main effects of condition, β = .89, *t* = 12.80, *p* < .001, reflecting overall faster responses in the control than in the gender classification condition (*M*s = 932 ms versus 1107 ms, *SD*s = 81 ms and 153 ms), and a main effect of quadratic morph level, β = -.36, *t* = -7.715, *p* < .001, reflecting overall slower responses to faces as a function of the extent to which they were blended (Fig 2). Critically, the latter effect was qualified by an interaction with condition, β = -.41, *t* = -5.91, *p* < .001, which was interpreted by re-running the regression analysis for each condition separately. These analyses indicated that gender classification time was a unique quadratic function of morph level, β = -.62, *t* = -8.211, *p* < .001, with slower responses for the intermediate blends. In contrast, in the control condition, response time was unrelated to any of the predictors, *p*s > .1.

#### Attractiveness

**Fig 3:**
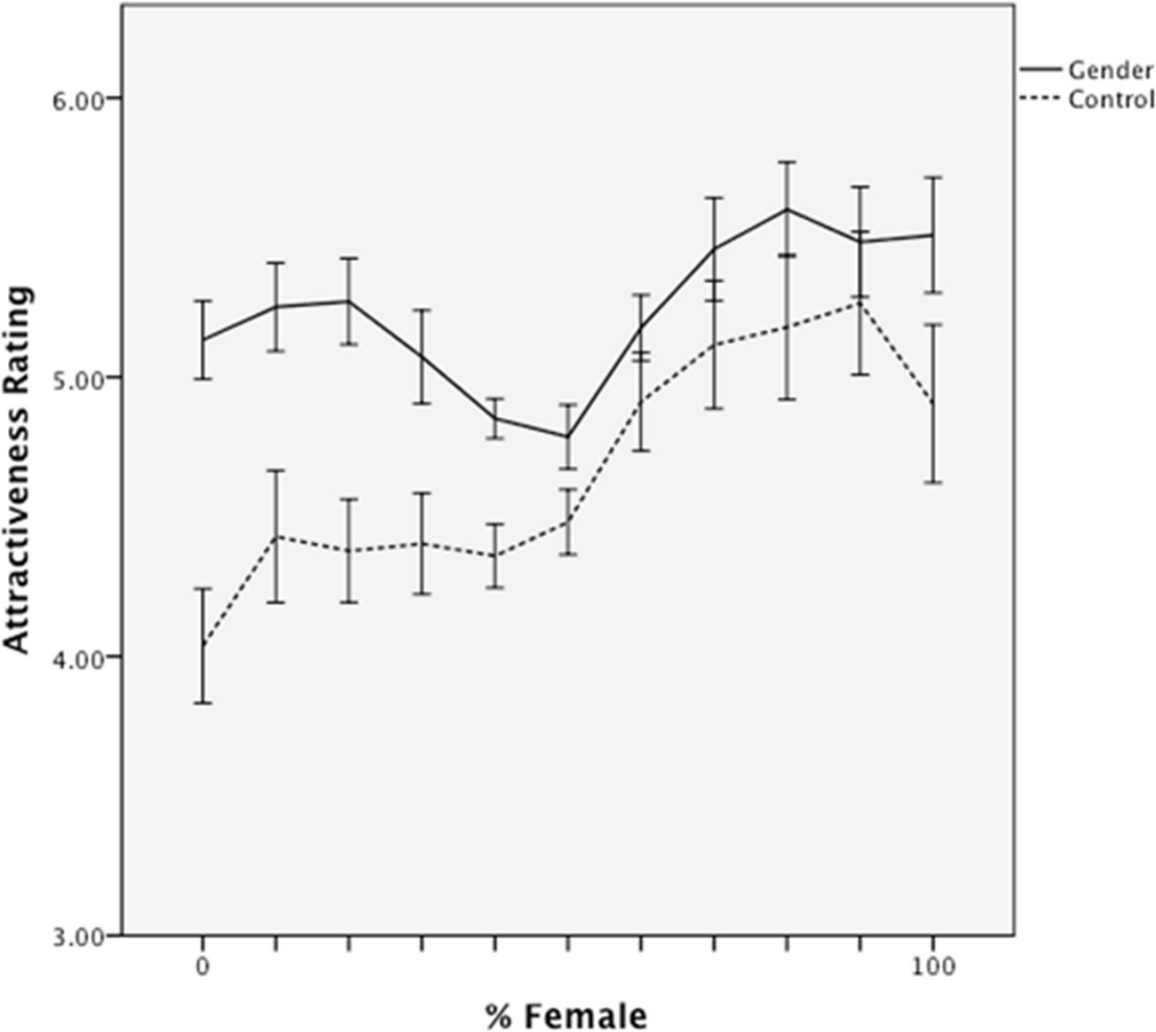
Mean attractiveness ratings as a function of morph level and classification group in Study 1. Error bars represent standard errors.

The same analytic strategy was taken with attractiveness judgments. The regression revealed main effects of experimental condition, β = .28, *t* = 3.22, *p* = .001, and morph level, β = .35, *t* = 6.10, *p* < .001. Participants who classified the faces by gender judged them as more attractive overall (*M* = 5.24, *SD* = .54) than controls (*M* = 4.68, *SD* = .75), and faces were more attractive as a linear function of the proportion of the female parent in the morph (Fig 3). There was also a condition by morph level interaction, β = -.15, *t* = -2.58, *p* < .05, due to the fact that “femaleness” is a weaker predictor of attractiveness in the gender classification condition, *r*(110) = .26, than in the control condition, *r*(110) = .47. Most importantly, a marginal interaction between condition and quadratic morph level emerged, β = .16, *p* = .06. The interaction is due to a “dip” in attractiveness in the gender classification condition, but not in the control condition (Fig 3). The difference in morph-attractiveness relationships was verified by running separate regressions for each experimental condition, which confirmed that the independent quadratic component was significant in the gender classification condition, β = .23, *t* = 2.51 *p* < .05, but not in the control condition, β = -.04, *p* > .5.

#### Mediation

The main analyses indicated that, as predicted, gender-blended faces were both more difficult to classify, and less attractive, than faces that were less ambiguous. To test the hypothesized role of classification fluency in the attractiveness effects, we conducted a mediation analysis with 10,000 bootstrap resamples using Hayes’ (2012) PROCESS procedure for SPSS [47]. The analysis indicated that the indirect quadratic effect of morph level on attractiveness, via classification time, controlling for the linear effect, did not include zero, *95% CI* [.014, .032]. Furthermore, the *direct* effect in the model was no longer significant *95% CI* [-.021, .004], suggesting that classification time fully mediates the attractiveness effects in the classification condition.

#### Discussion

In sum, the results of Study 1 support our hypothesis that the appeal of faces that blend features of both genders depends on how they are processed cognitively. According to our theoretical account, a mix of male and female facial features makes faces difficult to process, but only when gender classification is required, and in that case the resultant disfluency depresses a face’s attractiveness. Indeed, it is clear that compared to controls, participants who first categorized the gender-blended faces had difficulty doing so, and this difficulty explained a relative dislike for these faces, despite a preference for feminine features overall.

What is not clear, however, is whether gender classification *per se* is necessary to produce the effects. The control condition in Study 1 required only detection that the stimulus is a face–a low-effort task relative to gender classification, as reflected in faster response times. So, it could be argued that any task that requires sufficient attention to a stimulus with mixed features would result in dislike, perhaps because of general distaste for ambiguity. In contrast, we hypothesize that negative evaluations of gender-ambiguous targets is due to the difficulty in resolving relevant categorical membership. If so, asking participants to do another task that requires comparable attention to the stimulus, but does not make gender-blends selectively disfluent should not affect judgment. In Study 2, therefore, we added a second control condition that required classification by race, using a two-dimensional (gender x race) stimulus set. If the dislike of gender-ambiguous targets in Study 1 is due to gender-specific disfluency, processing time should increase, and attractiveness should decrease, only when faces are categorized specifically by gender.

## Study 2

### Method

#### Participants

One hundred and twelve psychology students from the University of Otago took part in the experiment in exchange for course credit. All participants identified as Caucasian or European decent. An additional 23 participants who identified as non-Caucasian participated in the study, but because of possible interactions with the race of the stimuli, these participants’ data were not analyzed.

#### Ethics statement

This project was approved by the ethics committee at the University of Otago. Participants provided written informed consent to participate in the studies

#### Stimuli

**Fig 4:**
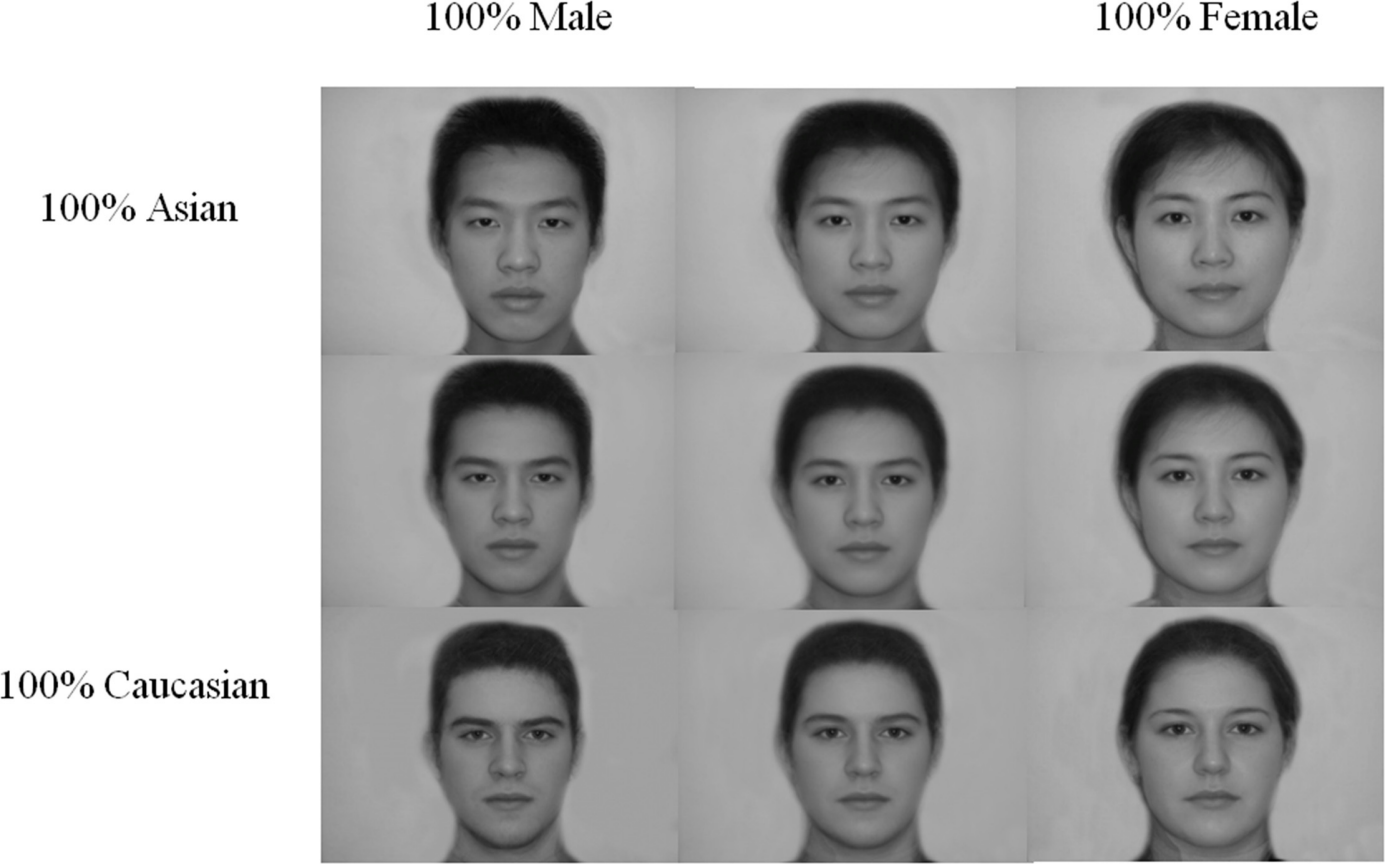
Examples of face blends used in Study 2.

A two dimensional stimulus set consisting of 100 face blends were created by first creating ten equal blends of Asian (Chinese) male and female parent faces, and ten equal blends of Caucasian male and female parent faces. The parent faces were gray-scale images that were each themselves averages of five distinct individuals of the same race and gender. Then, the first face on the Asian continuum (i.e., the 100% male / 0% female image) was morphed with the corresponding face on the Caucasian continuum, to create a new series of 10 morphs, each representing the same degree of “femaleness” but differing in “Caucasianness” to ten equivalent degrees. Following this procedure at each step in the two gender continua, we created 100 evenly spaced faces, each representing a unique racial and gender blend. All faces were 115x155 mm (examples appear in Fig 4).

#### Procedure

The procedure was identical to that of Study 1, except for the inclusion of a race classification condition. In this condition, prior to judging attractiveness, participants were instructed to categorize the face as either Asian or Caucasian, using appropriately labeled keys. Again, each face was preceded by a 1000 ms fixation cross (or, if the participant failed to respond within 2000 ms, a question mark) and replaced by the attractiveness scale as soon as the participant responded.

### Results and Discussion

#### Manipulation check

As in Study 1, trials in which response times were shorter than 200 ms and longer than 2000 ms were excluded from the analyses, as were all data of 29 participants who used incorrect response keys on the majority of trials and/or failed to classify at least 2/3 of the faces within the time constraints.

To verify that the morphing procedure had the intended effect on the perception of the faces’ gender, the proportion of participants in the gender classification condition who judged each image as female was regressed on the linear, quadratic, and cubic components of femaleness, controlling for Caucasianness. As in Study 1, the results revealed strong linear and cubic effects, β = 1.67 and -.82, *t*s = 27.01, and -13.24, *p*s < .001 (a weaker quadratic effect also emerged, β = .07, *t* = 2.73, *p* < .01). The model accounted for 95% of the variance in gender classification.

#### Fluency

**Fig 5:**
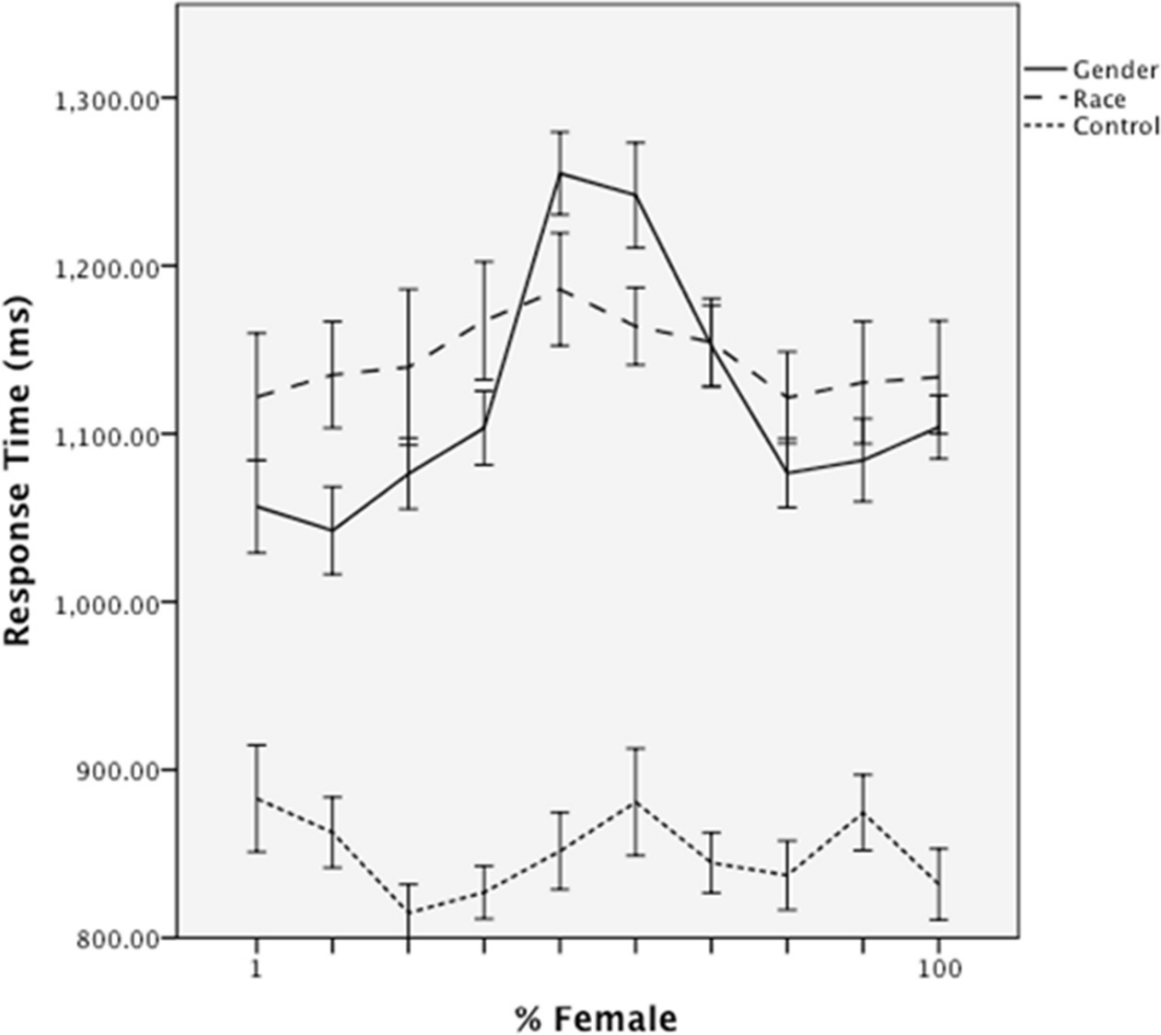
Mean classification response time as a function of morph level and classification group in Study 2. Error bars represent standard errors.

Classification times were regressed on experimental condition (dummy-coded to contrast the gender condition against the no-classification and racial classification conditions), linear and squared femaleness, and their interactions, controlling for race effects. The analysis revealed main effects of experimental condition, β = .42, *t* = 4.25, *p* < .001. Participants were slower at processing faces in the gender classification condition (*M* = 1119ms, *SD* = 102 ms) and the race classification condition (*M* = 1145 ms, *SD* = 107 ms) than in the no-classification condition (*M* = 865 ms, *SD* = 69 ms) (Fig 5). Note that the overall response times in the gender classification condition were very similar to the race classification condition, suggesting that the latter is a good control for overall attention to the faces.

Critically, a quadratic main effect of femaleness, β = -.17, *t* = -3.10, *p* < .005, was qualified by an interaction with experimental condition, β = -.18, *t* = -2.21, *p* < .05. Separate regression models revealed that the quadratic effect of femaleness was stronger in the gender classification condition, β = -.46, *t* = -5.51, *p* < .001, than in the race classification condition (β = -2.02, *p* < .05) or no-classification condition (β = .16, *ns*). In sum, as expected, faces with mixed gender features were strongly disfluent only when participants classified them in terms of their gender (Fig 5).

#### Attractiveness

**Fig 6:**
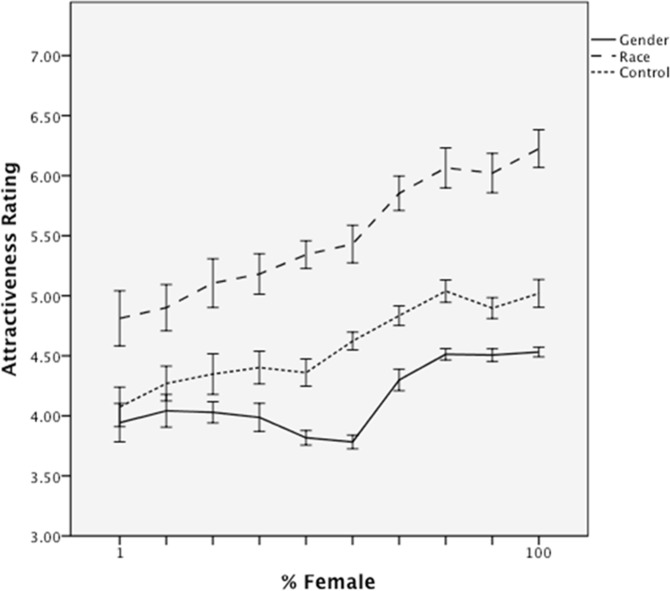
Mean attractiveness ratings as a function of morph level and classification group in Study 2. Error bars represent standard errors.

The results for attractiveness are plotted in Fig 6. The same regression model of attractiveness judgments revealed a main effect of experimental condition, β = -.66, *t* = -10.33, *p* < .001. Participants who classified the faces by gender rated them as overall less attractive (*M* = 4.15, *SD* = .40) compared to participants in the no-classification (*M* = 4.64, *SD* = .46) or race classification (*M* = 5.49, *SD* = .74) conditions. The attractiveness of faces also increased as a linear, β = .39, *t* = 10.75, *p* < .001, and quadratic, β = .08, *t* = 2.27, *p* < .05, function of their femaleness. Critically, both effects interacted with experimental condition, β = -.12, *t* = -3.40, *p* < .005, and β = .09, *t* = -1.79, *p* = .075. Separate regression models of linear and quadratic effects within each experimental condition revealed that although faces were preferred in all conditions as a linear function of their femaleness (*p*s < .001) the effect was weaker in the gender classification condition (β = .52) than in the no- classification (*b =* .64) or race- classification (*b =* .68) conditions. Most importantly, the quadratic component–the “dip” in attractiveness for gender-ambiguous targets–was significant only in the gender classification condition (β = .29, *p* < .001) and not in either control condition (β = -.004 and .04).

#### Mediation

Thus, as predicted, gender-blended faces were both more difficult to classify and were less attractive than less ambiguous faces. A mediation analysis using 10,000 bootstrap resamples indicated that the indirect quadratic effect of femaleness on attractiveness, via classification time, did not include zero *95% CI* [.002, .01]. In other words, the “dip” in attractiveness (Fig 6) was partially explained by the “peak” in classification effort (Fig 5).

#### Discussion

In sum, Study 2 closely replicates the key findings of Study 1. Again, participants found gender-ambiguous faces hard to classify, and the difficulty coincided–both qualitatively and statistically–with a distinct dip in their attractiveness, despite an overall preference for feminine features. Neither effect was evidenced when no classification, or classification on a gender-irrelevant dimension, was required. The latter finding, in particular, rules out the possibility that Study 1’s effects are due simply to greater attention to the stimuli (which, for example, highlights their ambiguous aspects). The results argue, instead, for a (dis)fluency mechanism, in which classification difficulty with respect to a specific stimulus dimension produces negative affect that weighs against the appeal of stimuli that are ambiguous on that dimension.

## General Discussion

Drawing on recent advances in fluency theory [43], we proposed an account that explains when, and why, gender ambiguity can lead to negative responses. Despite recent and dramatic changes in the gender categories available to describe ourselves and others, traditional gender prototypes for facial appearance are strong, and men and women who deviate from them can elicit cognitive disfluency in the perceiver. Because disfluency is negatively valenced, we predicted that gender-ambiguous individuals should be less positively judged. Critically, however, processing difficulty is more likely to occur when such individuals are judged in the context of gender categories.

Two studies provided strong support for this account. In both studies, when gender classification was not required, facial attractiveness was a simple linear function of the extent of its “feminine” features. However, requiring participants to classify a face as male or female prior to judging the face produced striking nonlinearities. Compared to more “pure” male or female faces, gender-mixed faces were less attractive (a U-shaped relation), as well as more difficult to classify (an inverse U-shape). Moreover, Study 2 showed that these effects are not merely a byproduct of greater attention being allocated to the faces in the gender classification condition. Requiring classification by another social dimension (i.e., race) did enhance overall time spent processing faces, but did not specifically reduce the fluency and attractiveness of gender-mixed faces. Most importantly, in both studies, the “dip” in the attractiveness of gender-ambiguous faces was statistically explained by cognitive effort in determining their gender, which presumably produced negative affect that transferred to the face itself.

It should also be noted that, while participants in the non-gender-classification conditions did not show the striking negative reaction to gender-ambiguous faces, neither did they show a *positive* reaction to these faces. This could be expected from the literature on the attractiveness of morphed faces, termed the “beauty-in-averageness effect.” Even though this is generally a replicable phenomenon [48], it is not an inevitable one [42], and we know little evidence for “beauty-in-gender averageness”. Furthermore, according to our model, the “beauty-in-averageness effect” should only emerge when blends function as a category prototype and thus become fluent (easier to process), with cognitive ease generating positive affect [49]. We did not obtain greater fluency of the blend in any of the control conditions, which may account for the lack of a “boost” in attractiveness for these blends. Also, it is possible that the pattern in the control condition is represents a form of schema-driven affect [50]. Specifically, assuming participants categorically prefer female to male features, the attractiveness of ambiguous faces may follow from the likelihood that they are classified as female.

Even though, in the current studies, gender-ambiguous targets suffered no negativity unless they were explicitly classified by gender, there are other situations in which individuals may be classified automatically, creating spontaneous disfluency even when classification is not required by the situation. For example, if a person has very strong or very narrow prototypes of male and female faces (perhaps as a result of exposure to a particular population or to particularly salient individuals), then seeing gender-atypical faces may spontaneously elicit classification disfluency. This is not unlike the spontaneous disfluency and discomfort that emerges when participants see faces representing blends of two celebrities (e.g., the well-known “Bushama” morph) [42].

At a meta-theoretical level, our results supplement the field’s nearly exclusive focus on ultimate explanations of male attractiveness, which explains discrepancies in the literature as a consequence of competing short-term and long-term mating goals. These explanations, while certainly part of the story, do not speak to the proximate mechanisms by which such goals are implemented cognitively. Processing fluency may provide one answer. For example, many studies have documented women’s preference for men with typically masculine traits (including facial and other bodily features) during the fertile phase of their menstrual cycles [29]. Our fluency account explains the mechanism of this preference, if one assumes that such ovulating women are more likely to categorize others in terms of their gender. As we have shown, the process of classification itself will produce a bias for men who are easiest to classify as such. Importantly, our account suggests that these effects emerge out of generic cognitive mechanisms linking categorization and affect; it does not appeal to any domain-specific mechanisms of optimal mate detection that is regulated by hormonal mechanisms.

Finally, we note that, although the current research is framed primarily in terms of “feminine men”, a perfectly complementary interpretation holds for “masculine women”. Although, as noted in the Introduction, the attractiveness of such individuals has been the subject of far less study, and no empirical controversy, our theoretical framework offers a cognitive account for why they are perceived negatively. Moreover, our account predicts circumstances in which such women will be judged *positively*, even if extant research has yet to identify such contexts empirically, and has the potential to unify the study of gender-ambiguity under a single set of proximate cognitive mechanisms.

## Supporting Information

S1 File Summary of multilevel modeling (MLM) results for Study 1 and Study 2. (DOCX)

S2 File Alternative analyses on the race-morphing dimension in Study 2. (DOCX)

S3 File Raw data from Study 1. (SAV)

S4 File Raw data from Study 2. (SAV)
