# Supplementary material for: Johnny Depp, Reconsidered: How Category-Relative Processing Fluency Determines the Appeal of Gender Ambiguity
Source: PLoS One. 2016 Feb 4;11(2):e0146328. doi: 10.1371/journal.pone.0146328 (PMC4742244; doi:10.1371/journal.pone.0146328)
Supplement: S1 File — (DOCX) [file pone.0146328.s001.docx]

**Supporting Information**

**Multilevel modeling (MLM)**

All repeated-measures analyses (including classification RTs and attractiveness ratings) used multilevel modeling (MLM) via maximum likelihood, since this method offers numerous analytical advantages — including more effective handling of unbalanced data with missing and/or non-independent observations, reliance on fewer assumptions regarding covariance structures, and increased parsimony and flexibility between models (Bagiella, Sloan, & Heitjan, 2000). All models were built using the *lme4* (Bates, Maechler, Bolker, & Walker, 2014) and *lmerTest* (Kuznetsova, Brockhoff, & Christensen, 2014) packages in *R*. To obtain p-value estimates for fixed effects, Type III Satterthwaite approximations were used, which can sometimes result in decimal degrees of freedom, based on the number of observations (West, Welch, & Galecki, 2014).

Final MLMs were selected based on top-down model building. First, models were created with a maximal factor structure, using all fixed effects and random intercepts for stimuli and participants (along with their interactions) that would allow for model convergence. Next, a nested model was trimmed by removing the random-effect that accounted for the least variance, and tested against the previous “full” model. If no significant difference was detected, the nested model was set as the new “full” model. This process was repeated until a significant difference was found between the “full” and nested models, at which point the “full” model was set as the final model to use for fixed effects testing. Significance statistics between model fits were determined by stepwise χ^2^ likelihood-ratio tests, via nested model comparison.

**Study 1.** On RTs, this MLM procedure was used on a fixed effects structure that included Condition (2 levels: control, gender-classification) and Male-Female Morph Level (11 values; herein called MF_morph_). MF_morph_ was modeled using continuous fixed effects for both the linear component (MF_morph_^1^) and quadratic component (MF_morph_^2^), as well as their respective interactions with Condition. Random effects for the maximal model included intercepts fit across subjects, stimulus face-pairs, and their interactions with fixed effects factors. Here, all effects were significant. We observed main effects on Condition, *F*(1, 71.91) = 39.13, *p* < .001, MF_morph_^1^, *F*(1, 118.44) = 43.65, *p* < .001, and MF_morph_^2^, *F*(1, 118.78) = 37.80, *p* < .001. Critically though, we also detected the predicted quadratic Condition x MF_morph_^2^ interaction, *F*(1, 667.76) = 108.87, *p* < .001, as well as the linear Condition x MF_morph_^1^ interaction, *F*(1, 663.83) = 112.38, *p* < .001 (see the main text for follow-up descriptions of these interactions).

For attractiveness ratings, we applied the same MLM strategy as Study 1 RTs. Once again, we observed the expected quadratic Condition x MF_morph_^2^ interaction, *F*(1, 727.67) = 13.43, *p* < .001, along with the linear Condition x MF_morph_^1^ interaction, *F*(1, 725.22) = 19.21, *p* < .001. Note that we also found a marginal main effect of Condition, *F*(1, 72.01) = 3.00, *p* = .088 (for breakdowns of these effects, refer to the main text).

**Study 2.** With Study 2, we used a similar MLM strategy, but we also incorporated two important changes (necessitated by the experimental design). First, the between-subject Condition fixed effect factor now contained three levels (i.e., control, gender-classification, and race-classification in Study 2) instead of two levels (i.e., control and gender-classification in Study 1). Second, since all 100 stimuli used in Study 2 were generated from the same parent faces, stimulus-level random effects were no longer modeled based on face-pair (but rather, on each individual stimulus).

For RTs, we applied a fixed effects structure that included Condition (3 levels: control, gender-classification, race-classification), MF_morph_ (10 values), and Asian-Caucasian Morph Level (10 levels; herein called AC_morph_). Similar to before, MF_morph_ was modeled using continuous fixed effects for both the MF_morph_^1^ (linear) and MF_morph_^2^ (quadratic) components, as well as their respective interactions with Condition. As was the case with Study 1, all effects were significant. Main effects emerged for Condition, *F*(2, 79.97) = 10.52, *p* < .001, AC_morph_, *F*(1, 109.53) = 35.25, *p* < .001, MF_morph_^1^, *F*(1, 97.61) = 17.87, *p* < .001, and MF_morph_^2^, *F*(1, 97.52) = 17.11, *p* < .001. Crucially though, we also saw the predicted quadratic Condition x MF_morph_^2^ interaction, *F*(2, 680.98) = 14.69, *p* < .001, along with the linear Condition x MF_morph_^1^ interaction, *F*(2, 682.30) = 15.90, *p* < .001 (see the main text for follow-up analyses of these effects).

On attractiveness ratings, we followed the same MLM procedure as Study 2 RTs. As before, we observed evidence that the quadratic male-female effect depended on Condition, through a marginal Condition x MF_morph_^2^ interaction, *F*(2, 745.28) = 2.92, *p* = .055. This was also accompanied by significance on the higher-order linear effect, via a Condition x MF_morph_^1^ interaction, *F*(2, 745.61) = 4.63, *p* = .01. Note that similar to Study 2 RTs, we also detected significant main effects on attractiveness for both Condition, *F*(2, 79.99) = 10.29, *p* < .001, and AC_morph_, *F*(1, 249.67) = 130.61, *p* < .001 (for more detail on these effects, refer to the main text).
