# Supplementary material for: Johnny Depp, Reconsidered: How Category-Relative Processing Fluency Determines the Appeal of Gender Ambiguity
Source: PLoS One. 2016 Feb 4;11(2):e0146328. doi: 10.1371/journal.pone.0146328 (PMC4742244; doi:10.1371/journal.pone.0146328)
Supplement: S2 File — (DOCX) [file pone.0146328.s002.docx]

**Supporting Information**

**Multilevel modeling (MLM)**

All repeated-measures analyses (including classification RTs and attractiveness ratings) used multilevel modeling (MLM) via maximum likelihood, since this method offers numerous analytical advantages — including more effective handling of unbalanced data with missing and/or non-independent observations, reliance on fewer assumptions regarding covariance structures, and increased parsimony and flexibility between models (Bagiella, Sloan, & Heitjan, 2000). All models were built using the *lme4* (Bates, Maechler, Bolker, & Walker, 2014) and *lmerTest* (Kuznetsova, Brockhoff, & Christensen, 2014) packages in *R*. To obtain p-value estimates for fixed-effects, Type III Satterthwaite approximations were used, which can sometimes result in fractional degrees of freedom, based on the number of observations (West, Welch, & Galecki, 2014).

Final MLMs were selected based on top-down model building. First, models were created using a maximal factor structure, using all fixed-effects and random intercepts for stimuli and participants (along with their interactions). Next, a nested model was trimmed by removing the random-effect that accounted for the least variance, and tested against the previous model. Significance statistics between model fits were determined by stepwise χ^2^ likelihood-ratio tests, via nested model comparison. This process was repeated until no significant difference resulted from this nested model comparison, at which point the final model was set for fixed-effects testing.

**Study 1.** On RTs, this MLM procedure was used with a Condition (2: control, gender-classification) x Male-Female Morph Level (11) fixed-effects structure. The random-effects for the maximal model included intercepts fit across subjects, stimulus face-pairs, and their interactions with fixed-effects factors. We observed the predicted Condition x Male-Female Morph Level interaction, *F*(10, 673.11) = 16.57, *p* < .001 (see the main text for a follow-up description of this interaction). Note that we also detected main effects for both Condition, *F*(1, 71.91) = 39.83, *p* < .001, and Male-Female Morph Level, *F*(10, 109.43) = 10.59, *p* < .001.

For attractiveness ratings, we applied the same Condition (2: control, gender classification) x Male-Female Morph Level (11) fixed-effects structure, using MLM testing. Once again, we observed the expected Condition x Male-Female Morph Level interaction, *F*(10, 723.47) = 2.90, *p* = .001 (for a breakdown of this interaction, refer to the main text). Further, we found a marginal main effect of Condition, *F*(1, 72.01) = 2.93, *p* = .09, and a significant main effect of Male-Female Morph Level, *F*(10, 162.44) = 8.45, *p* < .001.

**Study 2.** For Study 2, we used a similar MLM strategy, but with two important changes (necessitated by the experimental design). First, the between-subject Condition fixed-effect factor now incorporated three levels (i.e., control, gender-classification, or race-classification in Study 2) instead of two levels (i.e., control or gender-classification in Study 1). Second, since all 100 stimuli used in Study 2 were generated from the same parent faces, stimulus-level random-effects were no longer modeled based on face-pair (but rather, on each individual stimulus).

With RTs, we applied a Condition (2: control, gender classification) x Male-Female Morph Level (10) fixed-effects structure, using random intercepts fit across individual subjects and stimuli (along with their fixed-effect interactions). Similar to Study 1, we saw the predicted Condition x Male-Female Morph Level interaction, *F*(18, 7326.60) = 4.12, *p* < .001 (see main text for a follow-up analysis of this interaction). We also observed main effects for both Condition, *F*(2, 80.00) = 10.56, *p* < .001, and Male-Female Morph Level, *F*(9, 90.10) = 4.10, *p* < .001.

On attractiveness ratings, we followed the same MLM procedure as RTs. Once again, we observed the expected Condition x Male-Female Morph Level interaction, *F*(18, 729.05) = 2.06, *p* = .006 (refer to the main text for a breakdown of this interaction). Also similar to RTs, we detected significant main effects on attractiveness for both Condition, *F*(2, 79.99) = 10.14, *p* < .001, and Male-Female Morph Level, *F*(9, 142.50) = 8.20, *p* < .001.

**Supporting Information**

**Race blending effects**

Although race is not the focus of the current studies – race was manipulated only to make possible an alternative task to contrast with gender-classification – it is interesting to consider how race itself predicts attractiveness across the three classification conditions. Thus, we ran a parallel set of analyses, using *racial classification* (dummy coded), linear and squared “Caucasianness,” and their interactions, to predict fluency and attractiveness, controlling for gender ambiguity effects.

*Classification times (fluency)*

The analysis of classification times revealed main effects of experimental condition, *β *= .62, *t* = 6.75, *p* < .001; participants were slower at processing faces in the gender classification condition (M=1119ms, SD=102ms) and the race classification condition (M=1145ms, SD=107ms) than in the no- classification condition (M=865ms, SD=69ms). In addition, linear and quadratic main effects of Caucasianness, *β *= .22, *t* = 4.17, *p* < .001, and *β *= -.16, *t* = -3.16, *p* < .005, reflected the fact that more Caucasian faces were classified more slowly overall, and more racially ambiguous faces were classified more slowly than racially unambiguous faces. The latter effect interacted with experimental condition, *β *= -.52, *t* = -3.33, *p* < .001. Separate regression models revealed that the quadratic effect was significant only in the race classification condition, *β *= -.49, *t* = -8.40, *p* < .001, and not in the gender classification or control conditions condition, *β *= -.07 and *β *= .14.

*Attractiveness*

The attractiveness analysis revealed a main effect of experimental condition, *β *= .74, *t* = 15.79, *p* <.001. Participants who classified the faces by gender rated them as overall less attractive (M=4.15, SD=.40) compared to participants in the no- classification (M=4.64, SD=.46) or race classification (M=5.49, SD=.74) conditions. The attractiveness of faces also increased as a linear, *β *= .43, *t* = 16.43, *p* < .001, and quadratic, *β *= -.09, *t* = -3.36, *p* = .001, function of their Caucasianness, both of which interacted with experimental condition, *β *= .19, *t* = 7.22, *p* < .001, and *β *= -.08, *t* = -2.17, *p* < .05. Separate regression models of linear and quadratic effects within each experimental condition revealed that although faces were preferred in all conditions as a linear function of their Caucasianness (*p*s<.001) the effect was stronger in the race classification condition (*β *= .66) than in the no- classification (.52) or gender classification (.36) conditions. The quadratic effect appears to be due to a tapering off of the Caucasian bias toward the Caucasian end of the morph continuum (see Figure). The effect was significant only in the race classification condition (*β *= -.16, *p* < .001) and not in either control condition (*β *= -.05 and -.07).

*Mediation*

A mediation analysis using 10,000 bootstrap resamples indicated that the indirect quadratic effect of Caucasianness on attractiveness, via classification time, included zero (95% confidence intervals = -.003 and .005), and that, moreover, the direct effect remained significant when controlling for classification time. Essentially, this means there attractiveness judgments were not accounted for by differences in classification time for faces that were ambiguous on race.


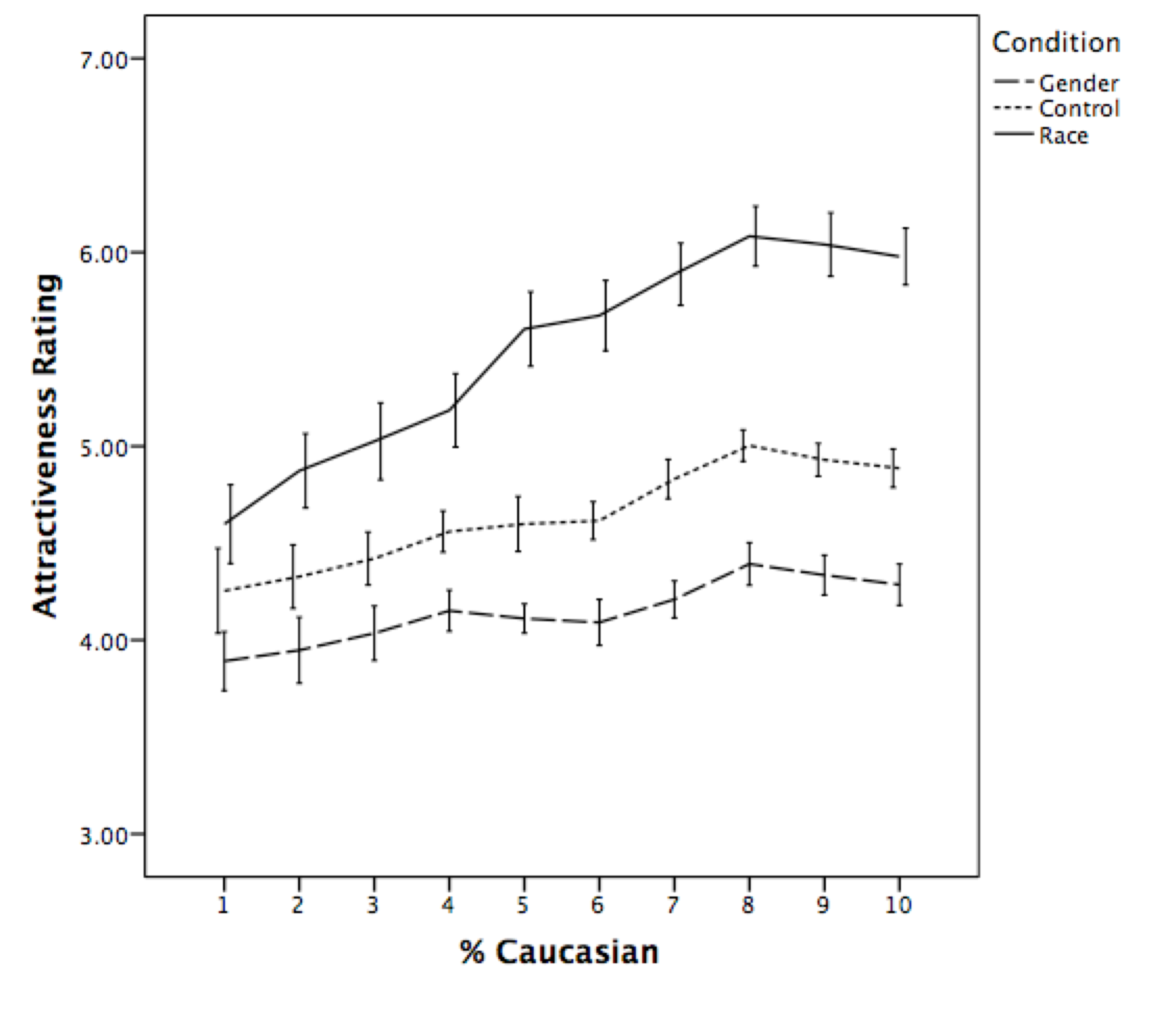


*Discussion*

The analyses of responses as a function of race blending (Asian – Caucasian) suggest that participants strongly relied on racial categories when making their attractiveness judgments. This may reflect participants’ own racial prejudices or some characteristics inherent to the stimulus set – a question that is orthogonal to the goals of current investigations. Critically, participants did not rely on the ease of classifying faces by race, as revealed by the absence of any fluency mediation. This is unlike attractiveness judgments as a function of gender dimension, which were highly sensitive to classification fluency, and consistent with our theoretical model. One possible reason for this difference is that our two-dimensional stimulus set (crossing gender and race) leads participants to adopt a simplistic judgment strategy for stimuli varying on race. In fact, it can be seen that in the race-classification condition, there is a very strong linear relationship between race continuum and attractiveness. In contrast, the relation between gender-continuum and attractiveness was weakest when participants focused on gender, suggesting that participants were not using stereotypical content, but instead relied on fluency. Future studies should investigate this interesting pattern, but it is known in the literature that in some circumstances participants will discount their fluency response and instead rely on stereotype content to determine their judgments (Schwarz & Clore, 2007).

*References*

Schwarz, N., & Clore, G. L. (2007). Feelings and phenomenal experiences. In E. T. Higgins & A. W. Kruglanski (Eds.), *Social Psychology: Handbook of basic principles* (2nd ed., pp. 385-407). New York: Guilford.
